# Monitoring Flow-Forming Processes Using Design of Experiments and a Machine Learning Approach Based on Randomized-Supervised Time Series Forest and Recursive Feature Elimination

**DOI:** 10.3390/s24051527

**Published:** 2024-02-27

**Authors:** Leroy Anozie, Bodo Fink, Christoph M. Friedrich, Christoph Engels

**Affiliations:** 1 Department of Computer Science, University of Applied Sciences and Arts (FH Dortmund), 44227 Dortmund, Germany; leroy.anozie@mailbox.org (L.A.); christoph.engels@fh-dortmund.de (C.E.); 2 WF Maschinenbau und Blechformtechnik GmbH & Co.KG, 48324 Sendenhorst, Germany; 3 Institute for Medical Informatics, Biometry and Epidemiology (IMIBE), University Hospital Essen, 45122 Essen, Germany

**Keywords:** flow forming, metal forming, multi-sensor, time series classification, design of experiments, recursive feature elimination, r-STSF, random forest

## Abstract

The machines of WF Maschinenbau process metal blanks into various workpieces using so-called flow-forming processes. The quality of these workpieces depends largely on the quality of the blanks and the condition of the machine. This creates an urgent need for automated monitoring of the forming processes and the condition of the machine. Since the complexity of the flow-forming processes makes physical modeling impossible, the present work deals with data-driven modeling using machine learning algorithms. The main contributions of this work lie in showcasing the feasibility of utilizing machine learning and sensor data to monitor flow-forming processes, along with developing a practical approach for this purpose. The approach includes an experimental design capable of providing the necessary data, as well as a procedure for preprocessing the data and extracting features that capture the information needed by the machine learning models to detect defects in the blank and the machine. To make efficient use of the small number of experiments available, the experimental design is generated using Design of Experiments methods. They consist of two parts. In the first part, a pre-selection of influencing variables relevant to the forming process is performed. In the second part of the design, the selected variables are investigated in more detail. The preprocessing procedure consists of feature engineering, feature extraction and feature selection. In the feature engineering step, the data set is augmented with time series variables that are meaningful in the domain. For feature extraction, an algorithm was developed based on the mechanisms of the r-STSF, a state-of-the-art algorithm for time series classification, extending them for multivariate time series and metric target variables. This feature extraction algorithm itself can be seen as an additional contribution of this work, because it is not tied to the application domain of monitoring flow-forming processes, but can be used as a feature extraction algorithm for multivariate time series classification in general. For feature selection, a Recursive Feature Elimination is employed. With the resulting features, random forests are trained to detect several quality features of the blank and defects of the machine. The trained models achieve good prediction accuracy for most of the target variables. This shows that the application of machine learning is a promising approach for the monitoring of flow-forming processes, which requires further investigation for confirmation.

## 1. Introduction

Flow forming is a metal forming process for the production of rotationally symmetrical parts such as flanged components and pressure cylinders [1,2]. In a flow-forming process, a blank, i.e., a short and thick metal disk, is mounted on a rotating spindle and stretched out by mechanically guided rollers [3]. In the process, the workpiece becomes longer and its wall thickness thinner, while the inside diameter remains constant [3].

**Figure 1:**
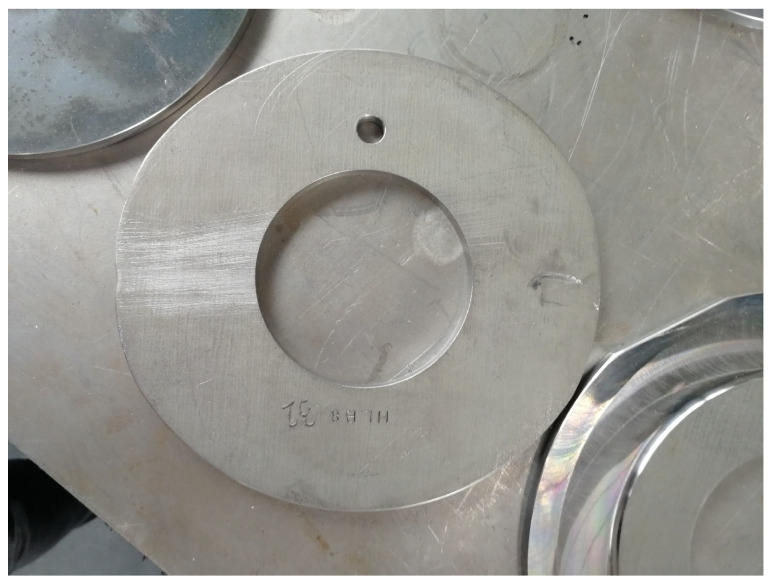
Ronde (Circular metal blank placed in the machine for processing).

**Figure 2:**
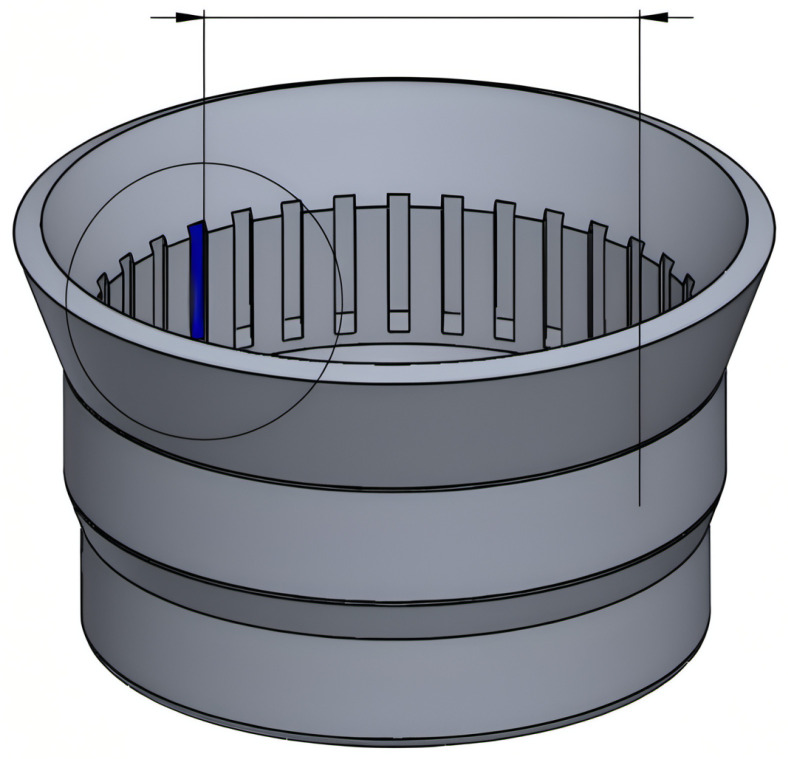
Technical drawing of the workpiece showing its toothed inner side.

**Figure 3:**
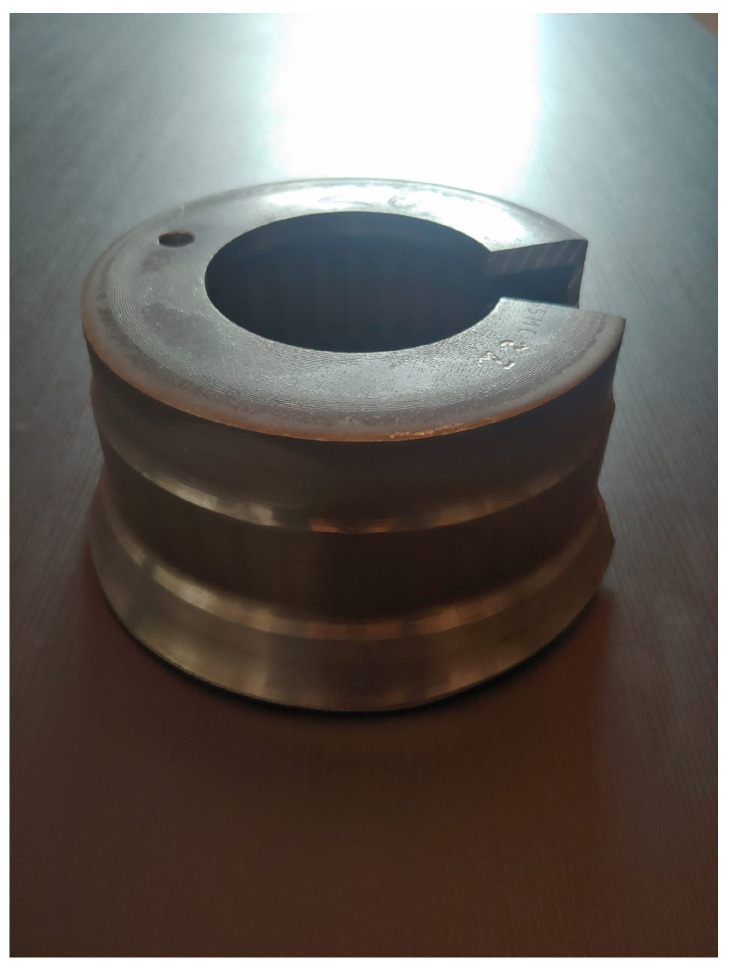
Product of the studied forming process. The missing piece on the right edge was subsequently milled out in order to be able to perform measurements on the workpiece.

**Figure 4:**
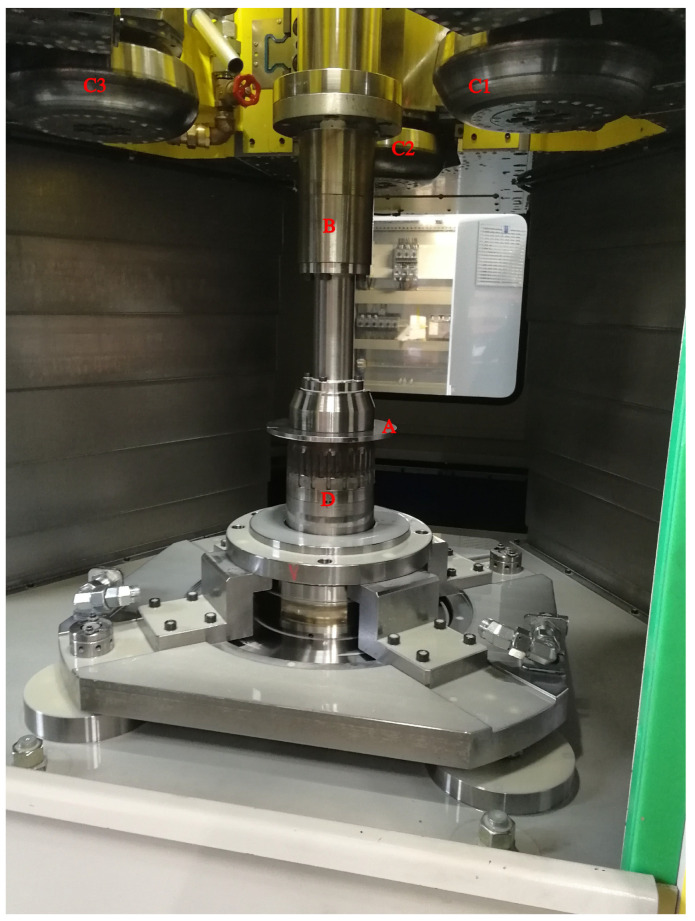
Interior of the VSTR 400-3S. Among other things, the fixed metal blank (marking A), the tailstock (marking B), the rollers (marking C1, C2 and C3) and the punch (marking D) are visible.

In the flow-forming process studied in the work at hand, a test machine, the VSTR 400-3S built by WF Maschinenbau, forms a circular metal blank (see Figure 1) in the gear component shown in Figure 2 and Figure 3. First, the blank is placed on the spindle of the machine (see Figure 4, marking A). When the program is started from the control panel, the tailstock (see Figure 4, marking B) then moves in from above and fixes the blank on the spindle. The three rollers of the machine (see Figure 4, marking C1, C2 and C3) are responsible for the actual forming. During the process, they move along a programmatically defined path, while the blank is rotated by the spindle. The free-spinning rollers are pressed against the rotating workpiece along the axial feed axis, which also causes them to rotate and deform the workpiece. The shaping in the inner part of the workpiece with the toothing shown in Figure 2 is defined by the punch (see Figure 4, marking B). During the entire forming process, the workpiece is cooled with a cooling liquid, which is why this is also referred to as cold forming.

The quality of the produced workpieces is highly dependent on the conditions or input variables of the forming processes, which primarily include the quality of the metal blanks used in the process and the preconditions of the machine itself. Blanks with an inexpedient geometry, such as an irregular thickness or a hole that is displaced from the center, can lead to undesired properties in the resulting workpiece, such as being asymmetrical. The causes of blanks with such inexpedient geometry can be errors or misconfigurations in the manufacture of the blanks as well as the inadvertent use of incorrect blanks. Similarly, machine defects can also lead to undesirable process results, such as irregularities on the surface of the finished workpiece caused by a worn out roller. The resulting waste of time and material increases as long as these problems go unnoticed. This is particularly problematic if the feeding of the machine is automated, which is not the case for the test machine used in this study, but may be the case for other flow forming machines as well as extensions of the VSTR 400-3S. Therefore, automated process monitoring capable of detecting and responding to such defects by issuing warnings, recommending actions, or even stopping the machine can prevent significant financial loss.

However, the mechanics of flow-forming processes, are not fully understood [4], so it is not possible to model them completely physically in order to implement the required monitoring functions on this basis. Consequently, data-driven modeling using machine learning algorithms is used to automatically monitor the process and the machine.

Machine learning is a branch of artificial intelligence that is widely used in business, medicine, industry and other fields [5]. Machine learning methods can be used to identify complex relationships in existing data sets that cannot be captured by “handcrafted” rules [6]. The machine learning algorithm used in this work is random forest (see, e.g., [7]). In so-called supervised learning methods like random forest, a training data set is used to model the relationship between attributes (also termed "features" or "variables") of considered objects and a defined target variable. The resulting model can then be used to determine the target value for new or unseen objects [5]. Random forests can be used for both classification (i.e., discrete target variables) and for regression (i.e., continuous target variables) [7].

Flow forming has been the subject of various studies, many of which have focused on modeling the relationships between process input parameters and the geometry of the finished workpieces (see Section 2.4). To the author’s knowledge, however, there is no literature describing approaches to data-driven monitoring of flow-forming processes to date. Furthermore, in the literature on the monitoring of other machine processing operations, prediction of process input variables is a rarely considered topic and the time series data used are mostly one-dimensional, which leaves possible potentials such as monitoring the machine using multiple sensors unaddressed.

The work at hand addresses these research gaps and proposes an approach for the acquisition and processing of sensor (and other) data as well as for the generation of machine learning models that predict process input variables using this data. The contributions of this work can be summarized as follows:

- Demonstration of the feasibility of utilizing machine learning and sensor data to monitor flow-forming processes.
- Development of a practical approach to monitor flow-forming processes. The approach includes an experimental design capable of providing the necessary data, as well as a procedure for preprocessing the data and extracting features that capture the information needed by the machine learning models to detect defects in the blank and the machine.
- Development of a feature extraction algorithm for multivariate time series classification, derived from the state-of-the-art algorithm r-STSF (see [8]), incorporating extensions for multivariate time series and metric target variables.

## 2. Related Work

### 2.1. Condition-Based Maintenance

Over time, machinery equipment suffers from wear and tear, which can lead to failures [9]. In many cases, e.g., because of safety risks or reduced productivity, such failures are unacceptable. Therefore, attempts are often made to prevent these failures through preventive maintenance measures [9]. In addition to the comparatively uneconomical approach of performing maintenance measures on the basis of a fixed schedule (calendar-based maintenance), there is condition-based maintenance or CBM for this purpose [9]. The aim of CBM is to monitor the condition of a machine by collecting data about it (condition monitoring or CM) and, based on this, to enable and carry out needs-oriented maintenance measures [9].

A variety of machine learning algorithms have been used for condition monitoring in manufacturing. Among others, commonly used are SVMs (see, e.g., [10,11,12,13]), artificial neural networks (see, e.g., [10,11,14]) and random forests (see, e.g., [10,13,15]). Comparisons of several of these algorithms in different condition monitoring applications are conducted in [14] (detection of faulty cycles of a cutting machine), [10] (classification of defects in ball bearings), [15] (detection of defects in robot arms) and [12] (classification of defects in vehicle transmissions). They show that, in this field, the results obtained with different learning algorithms usually differ only slightly, so that the choice of algorithm is not a high priority.

### 2.2. Data Acquisition

To obtain data for the training of machine learning models, data from production operations (see [15,16]) as well as from experiments conducted specifically for this purpose can be used. When conducting experiments, the Design of Experiments (DoE) methodology can be used to increase the effectiveness of individual experiments in order to reduce the number of experiments required, thus saving time and reducing costs [17]. Consequently, DoE is an integral part of the guideline for the development of monitoring systems for machining processes described in [17]. However, in practice, instead of using DoE, in practice, experiments are also often performed by testing the defects or unacceptable conditions one at a time in one or more experiments (see, e.g., [12,18]) or by running manufacturing processes until a defect occurs. The latter approach is used in cases such as [19,20], where the aim is to detect different stages of wear, and also in cases such as [21,22], where the run-to-failure data are used to predict the remaining time until a component breaks (Remaining Useful Life, RUL).

### 2.3. Sensors

**Figure 5:**
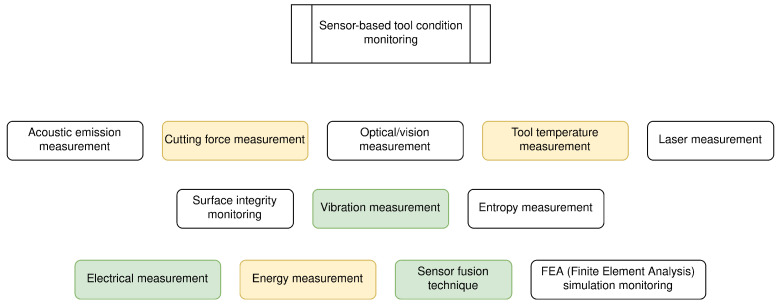
Commonly used sensor techniques in Tool Condition Monitoring. Sensor techniques used in the common work are marked in green; sensor techniques used indirectly or in a similar way are marked in yellow (Adapted from [24]).

The sensors used to monitor manufacturing processes can be of various types. These include, but are not limited to force, vibration, current and acoustic emission sensors [17]. Figure 5 shows an overview of the most common sensor technologies used in condition monitoring of manufacturing tools (Tool Condition Monitoring). Force sensors are particularly common [17,23]. The works [17] (machining), [24] (machining), and [23] (milling) deal with the different types of sensors, their advantages and disadvantages and their respective applications. In [13], a comparison is made of the accuracy achievable with different sensors individually, as well as with sensor fusion (feature set assembled from the signals of all sensors [13]), when classifying defects on the tools of an ultrasonic metal welder.

### 2.4. Monitoring Flow Forming Processes

While the monitoring of other manufacturing processes has been extensively studied in the literature, little work exists to date on the monitoring of flow-forming processes and, more generally, incremental rotary forming processes. This is also noted in [2,4]. To the author’s knowledge, there is currently no literature on data-driven process or condition monitoring of flow-forming processes (including CBM, TCM and Predictive Maintenance), particularly using machine learning-based methods.

Papers [2,4] lay some groundwork in this area by investigating correlations between intentionally provoked process failures and changes in sensor data (acoustic, vibration, ultrasonic) recorded during the processes. The goal here is to evaluate the potential of different approaches to monitoring flow-forming processes and incremental rotary forming processes [4], but not to implement data-driven condition monitoring.

Furthermore, there are several works that deal with predicting the geometry of the finished workpiece based on the process input parameters such as the machine settings. For instance, in [3], linear and quadratic models are used to predict ovality based on roller feed, spindle speed, and radius of the roller. In [25,26], the researchers train artificial neural networks to predict various geometry variables based on process inputs and compare them with linear models, with the neural networks producing significantly better results in both cases. Both [25,26] also attempt to optimize the process input parameters to induce a desired geometry in the finished part. In [25], this is achieved using particle swarm optimization and composite desirability, while in [26], the authors trained neural networks to approximate the input parameters necessary for a desired geometry. An approach developed in [27] allows for manipulation of the forming process during its execution to achieve a desired geometry. For this purpose, physical models, a laser to measure wall thickness, and a sensor for indirect estimation of material properties are used. Analyses of the relationships between the process input parameters and the forces acting during flow-forming processes can be found in [28,29,30].

## 3. Materials and Methods

### 3.1. Experimental Setup

**Figure 6:**
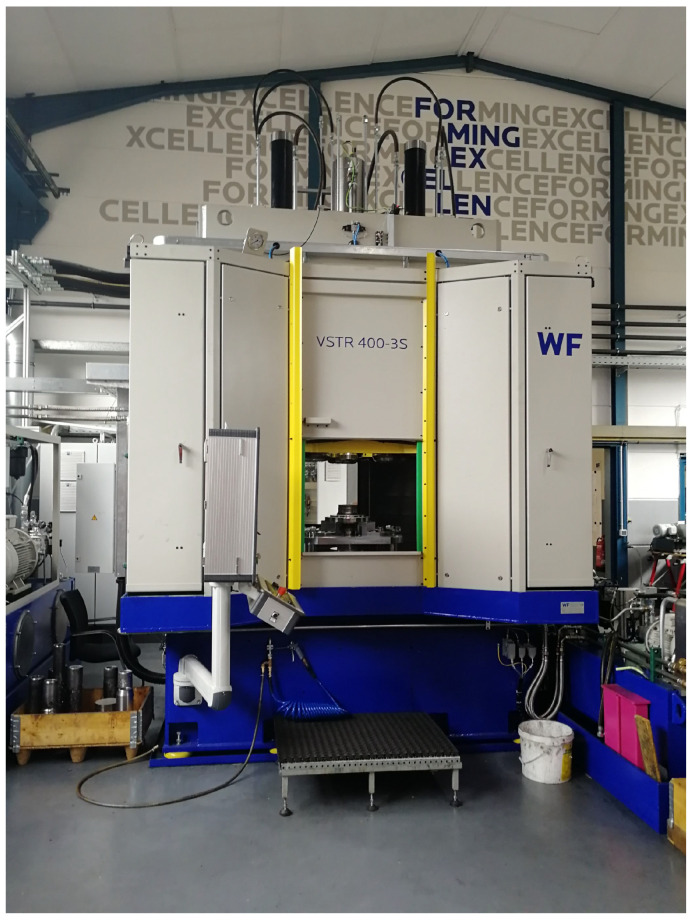
Front side of the VSTR 400-3S.

The data for the training and evaluation of the machine learning models are generated in experiments, which are carried out on the VSTR 400-3S (see Figure 6). The VSTR 400-3S is a machine that was built and specially modified for these experiments by WF Maschinenbau. It uses a rotating spindle and three mechanically guided rollers to form gear components with up to 400 mm in diameter. This process is called a flow-forming process [3] and is controlled by a CNC (Computerized Numerical Control) program, which contains the commands to operate the machine. The same CNC program is executed in all the experiments. Thus, the CNC program defines the example flow-forming process under study.

**Figure 7:**
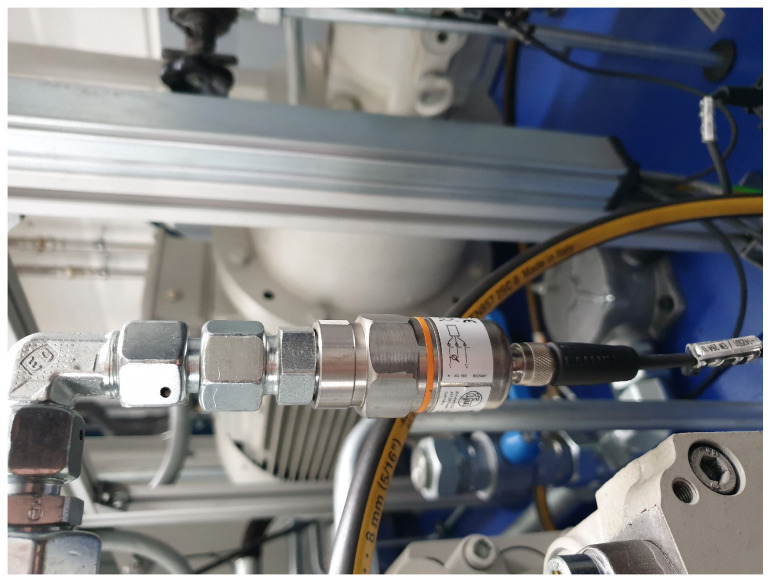
Sensor for measuring the pressure on one side of a hydraulic oil cylinder. A total of ten sensors of this type are installed in the machine. These are distributed on each of the two sides of the hydraulic oil cylinders of the three mechanically guided rollers and the spindle as well as one each for the hydraulic oil cylinders of the tailstock traverse and the ejector.

Multiple sensors installed in the machine collect several types of data during the forming process, such as pressure, vibration and temperature data. Figure 7 shows an example of a pressure sensor that measures the pressure on one side of a hydraulic oil cylinder. A complete list of all relevant sensors and signals can be found in Appendix A. Process monitoring using sensors is particularly challenging in flow-forming processes, mainly because both the workpiece and the processing tools, i.e., the rollers, rotate, making it impossible to place, for example, a vibration sensor directly at the site of deformation impossible. This is different to most other Tool Condition Monitoring applications, which typically involve a tool and a workpiece with one of them moving while the other is stationary and can be used to mount these sensors on. Additionally, in flow-forming processes, the area of contact between the tool and the workpiece is constantly moving and can change shape during the process, exacerbating the problem [4]. Because of these constraints, setups, like those, for example, in [31], who placed a vibration sensor directly on the tool to monitor turning operations, or in [32], who integrated a force sensor into a shear-cutting tool, cannot be implemented. The same applies to the approach of mounting the workpiece on the force sensor, as is carried out, for example, in [33] on a milling machine. Instead, to monitor flow-forming processes, sensors such as vibration sensors need to be placed further away from the source of the signal, which is a detrimental because this way, the system suffers more from noise introduced by vibrating components as well as loss of signal strength between components, both of which increase as the distance between the site of deformation and the sensor increases [4]. On the VSTR 400-3S, the tailstock traverse (common support beam for all three rollers) and the spindle bearing were chosen as locations for the vibration sensors with reasonable proximity to the deformation site, but without the need for significant modifications to the machine. The in-process forces applied to the workpieces are approximated using the pressures measured at the hydraulic oil cylinders and the physical model of the machine. A total of ten pressure sensors are used, one on each of the two sides of the hydraulic oil cylinders for the three mechanically guided rollers and the spindle, as well as one each for the hydraulic oil cylinders of the tailstock traverse and the ejector.

The sensor data are supplemented by the programmable logic controller (PLC), i.e., the control unit of the machine, with additional values such as position data of the rollers. The PLC, a Simatic S7 from Siemens, on the one hand, controls the movements of the machine by translating the CNC program into precise machine commands and sending them to the machine. On the other hand, it monitors the sensors and stores their signals at regular intervals. Since not all signals are recorded at the same sampling rate, interpolation of intermediate values is required for the signals with slower sampling rates. For this purpose, Akima splines are used, which is a mathematical method for fitting a “soft” and “natural” curve through a given set of data points [34].

### 3.2. Experiments

Two series of experiments are carried out, both designed using DoE. These experimental designs determine the variation of influencing factors, such as a defect in the machine or a changed geometry of the blank for the individual experiments. In this way, changes in the forming process are provoked that the machine learning models will later be supposed to detect in the sensor data.

**Figure 8:**
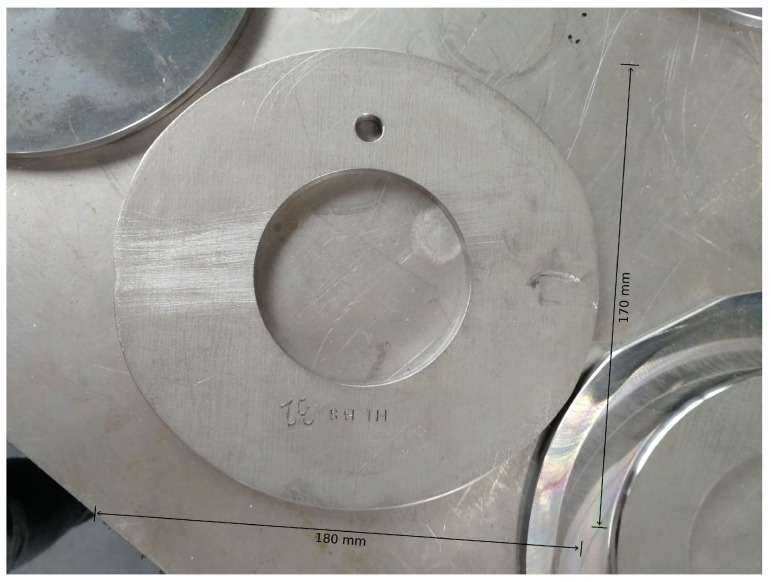
Metal blank with by grinding irregularly reduced diameter (change in influencing factor 3).

**Figure 9:**
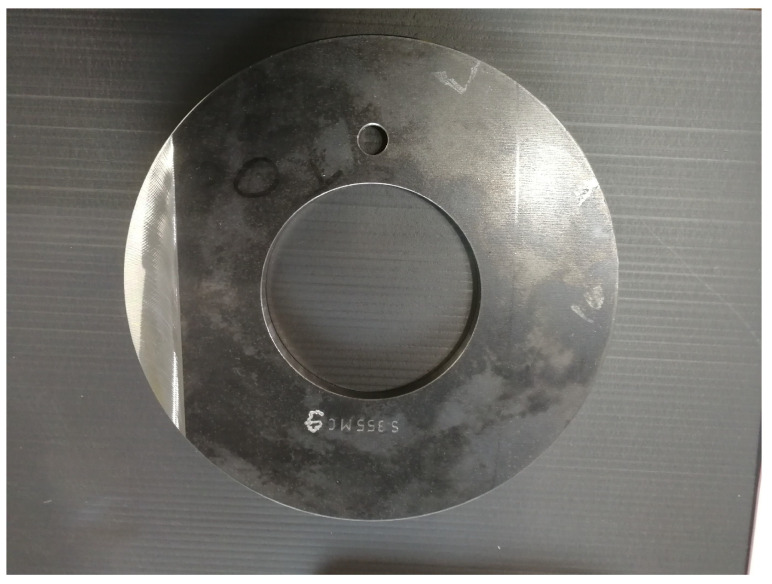
Metal blank with by grinding locally reduced thickness (change in influencing factor 4).

**Figure 10:**
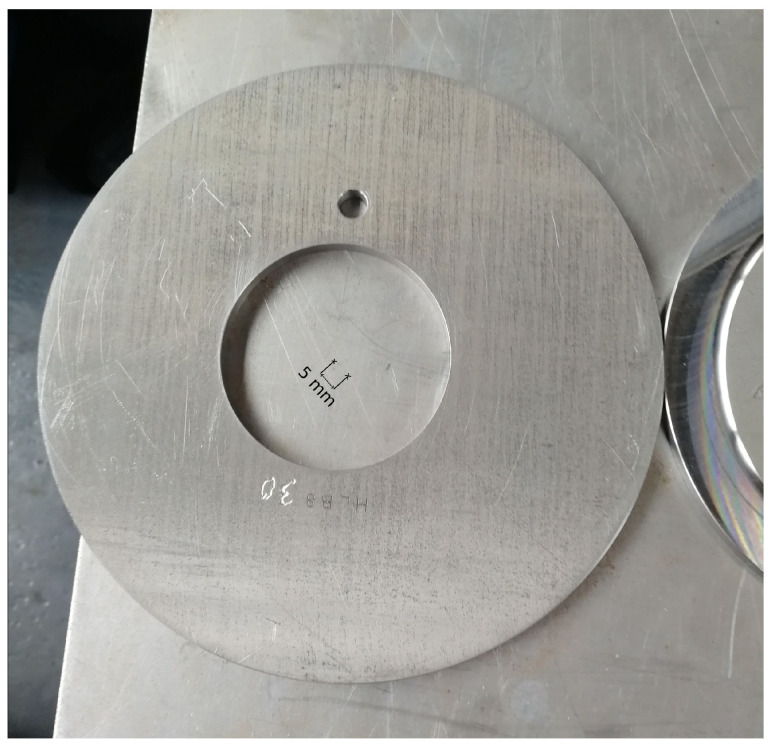
Metal blank with a hole of which the center deviates from the center of the blank (change in influencing factor 6).

**Figure 11:**
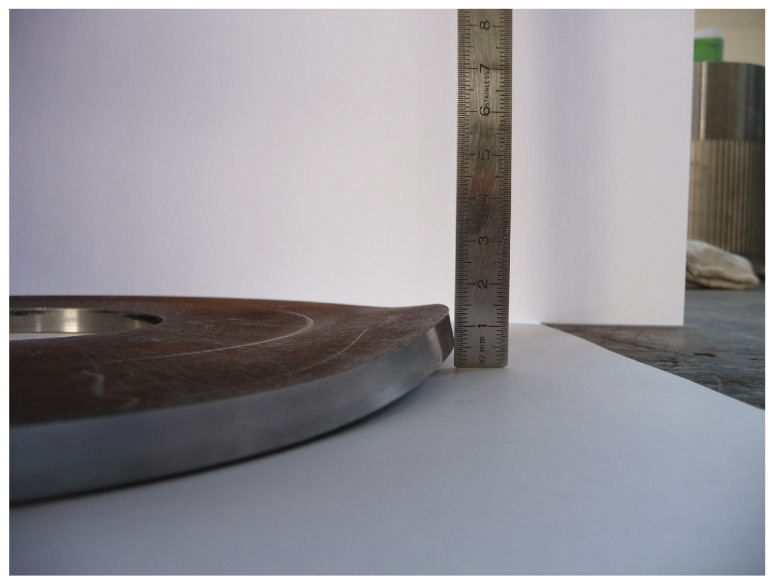
Metal blank with a defined deformation where the blank is not completely flat (change in influencing factor 7).

**Figure 12:**
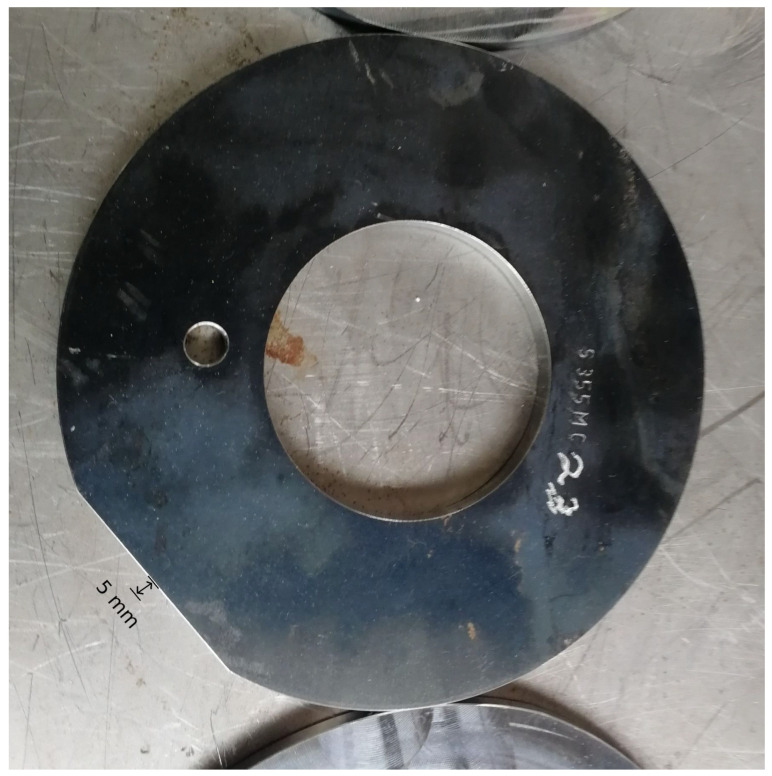
Metal blank with straight piece milled from it (change in influencing factor 8).

**Figure 13:**
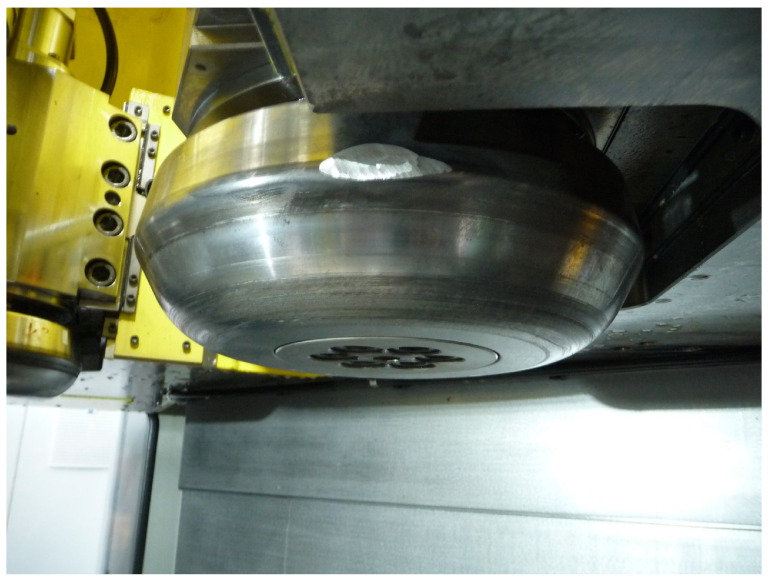
Roller that was damaged by grinding (change in influencing factor 10).

In the course of the experimental series, a total of ten factors (influencing variables) of the forming process, selected by the process experts at WF Maschinenbau, are varied. These include eight blank parameters, which are set by preparing the blanks, and two machine parameters, which can be set by making changes to the machine. The following parameters are investigated in the experiments:

1. Diameter of the blank;
2. Thickness of the blank;
3. Irregular diameter reduction in the blank by grinding (see Figure 8);
4. Local thickness reduction in the blank by grinding (see Figure 9);
5. Alloy from which the blank is made;
6. Deviation of the center of the hole from the center of the blank (see Figure 10);
7. A defined deformation of the blank where the blank is not completely flat (see Figure 11);
8. Straight piece milled from the blank (see Figure 12);
9. Flow rate of the cooling liquid;
10. Damage to one of the processing rollers by grinding (see Figure 13).

For all factors (except the alloy of the blank, where two different alloys were tested, which have different properties, while both being feasible for the process), there is an ideal or nominal value defined by the process experts. The deviations from these nominal values generated by preparation or configuration simulate causes for suboptimal process results occurring in practice.

### 3.3. Experimental Design I

The plan for the first series of experiments is based on a two-level experimental design, i.e., each factor is tested on two settings or levels, with 16 experiments. The plan is constructed according to the method of Plackett and Burman and extended to 32 experiments by a foldover. This foldover, which is a plain copy of the original experimental design with simultaneous inversion, increases the resolution of the experimental design from level III to level IV [35]. This means that the main effects are much less aliased and the design is suitable for determining main effects reliably [35]. The primary purpose of this experimental design is to screen the factors. It provides a basis for the pre-selection of factors for the second series of experiments, in which these factors are examined with a higher number of experiments. The two levels examined per factor correspond to the respective minimum and maximum values for the numerical factors. This results in large distances between the tested levels, which is particularly advantageous in early investigation phases [35]. The factor of the alloy of the blank is set to two alloys common for the produced component. The remaining factors are binary and each has a defective and a non-defective setting.

Additionally, the experimental design includes six so-called reference points, resulting in a total of 39 experiments for this experimental design. Reference points are experiments that have identical factor settings corresponding to the nominal values and thus represent the reference process. Due to the identical factor settings, these experiments allow the experimental system to be monitored for changes over time [35] and to estimate experimental dispersion [35].

The order of the experiments is generally randomized to avoid systematic errors (see [35]). Deviating from this, the sequence contains later adjustments that minimize the setting changes of the two factors which are concerned with the condition of the machine, i.e., factors no. 9 and 10. These adjustments are necessary due to the high effort of the setting changes of these factors.

### 3.4. Experimental Design II

The second experimental design contains six of the original ten factors, no. 1, 2, 6, 7, 9 and 10. The main criteria for this selection decision, made in consultation with the process experts, are the expected practical relevance of the individual factors and their influence on the forming process observed in the first series of experiments.

Experimental design II is composed of a single-factor foldover (copy of the experimental design with inversion of one of the factors [36]) of experimental design I (32 experiments), an adapted face-centered central-composite design (22 experiments), “single-defect experiments” (13 experiments), additional random experiments (16 experiments), and reference experiments (10 experiments). A total of 93 experiments are performed in the second series of experiments.

With the help of the single-factor foldover, the alias chains between two-factor interactions remaining after the first foldover in experimental design I are eliminated. The Central-Composite-Design contains eleven experiments and includes the factors 1, 2 and 6, while the remaining factors remain in the nominal state. This decision is based on the screening of the factors in the first series of experiments. The blank for the central point experiment is prepared to a diameter of 200 mm (for practical reasons this slightly different value is used instead of the correct mean value for the diameter, 199 mm), a thickness of 7.25 mm and a displacement of the hole of 2.5 mm. Since the two-levelled part of the experimental design already exhausts the minimum and maximum limits of the factors, a face-centered central composite design is used. In addition, for the two factors diameter and thickness, further points are added between the respective star points and the central point, so that the factor space is sampled even more tightly in these two dimensions using five levels each. All experiments of the face-centered central composite design are repeated twice each. Ten additional experiments serve as reference points. Furthermore, there are 13 single-defect experiments, i.e., experiments that deviate from the nominal values in only a single factor, thus posing a special challenge for models for target variable-independent detection of deviations from the nominal process. The remaining 16 experiments are randomized experiments in which the factor settings are generated entirely by a pseudorandom number generator. Experimental design II is also randomized as far as possible.

### 3.5. Training Pipeline and Test Pipeline

The individual steps of preprocessing and model training are embedded in two pipelines, i.e., defined processing sequences—one for training the models and one for evaluating new data or test data. Each data point represents a single experiment. To avoid data leaks, training and test data are separated as early as possible and all processing steps are performed separately on the partial data sets. The evaluation method used in the present work is repeated k-fold cross-validation, so that the training pipeline is used for the k training data sets and the test pipeline is used for the k evaluation data sets created in each repetition of the cross-validation. However, the pipelines are designed to be agnostic to the sampling method, so other methods such as hold-out sampling or cross-validation could also be used with these pipelines.

**Figure 14:**
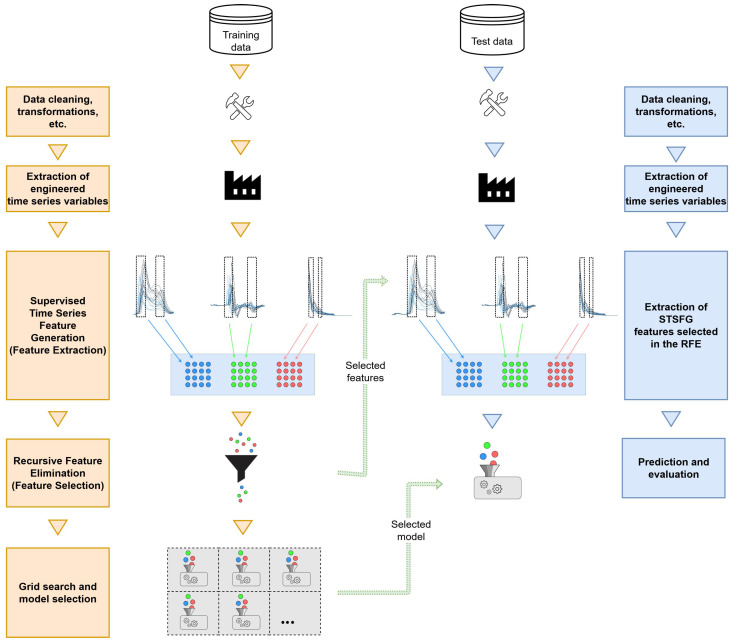
Training pipeline (**left**) and test pipeline (**right**). The training pipeline is used for the k training data sets and the test pipeline is used for the k evaluation data sets created in each repetition of the cross-validation. The different coloured dots represent the different features passing through the pipeline.

The training pipeline consists of the steps initial data preparation, generation of additional “hand crafted” time series variables, a feature extraction for multivariate time series, feature selection, and model generation and model selection. In the first step of the training, the data are cleaned and transformations of time series variables are performed (see Section 3.5.1). This is followed by the construction of additional time series variables that are assumed to be able to support the predictions of the models (see Section 3.5.2). After this, interval features are generated on all time series variables using the feature extraction algorithm developed later in Section 3.5.3 for multivariate time series, STSFG. The set of interval features is then reduced using Recursive Feature Elimination (see Section 3.5.4). Lastly, a grid search is performed on the resulting feature set, in which the hyperparameters of the random forest algorithm are optimized. The left side of Figure 14 illustrates the flow of this pipeline.

The test pipeline flows in a generally similar way as the training pipeline. First, the data are cleaned and transformed. Then, the defined time series variables are generated and added to the data set. In contrast to the training pipeline, where STSFG feature extraction followed by feature selection is performed next, the next step here is generating interval features, but only considering those features selected during the training pipeline’s feature selection. The final step is to evaluate the final random forest model on the test data. The final model is trained on the complete training data set and its hyperparameter settings correspond to the result of the grid search of the training pipeline. The right side of Figure 14 visualizes the flow of the test pipeline.

#### 3.5.1. Data Cleaning and Transformations of Time Series Variables

In order to improve the data quality and to prepare the data for the machine learning algorithms, several data cleaning and transformation operations are performed on the time series variables:

- Extraction of in-process data: The raw data set of each experimental series consists of the continuous time series of all the time series variables over the complete course of the experimental series, including the time between experiments. Using the position data of the rollers, the data recorded during the actual forming processes are separated from the data recorded while the machine is idle. The latter are discarded.
- Removal of empty runs: If the CNC program is executed without a blank being inserted (which sometimes needs to be carried out for different reasons), the corresponding data are discarded.
- Removal of not useful time series variables: Some of the time series variables supplied by the PLC are redundant. These are discarded.
- One hot encoding: Some of the time series variables supplied by the PLC are nominal scaled. These are transformed using one-hot encoding.
- Imputation of missing data points: Missing data points are imputed using LASSO (Least Absolute Shrinkage and Selection Operator, see [37]) regression.
- Merging the data from the PLC with the DoE data: The time series data supplied by the PLC are merged with the corresponding influencing variables from the experimental design. For this purpose, a consecutive process counter from the PLC data and the experiment number from the experimental design are used. The assignment between the process counter and the associated experiment number is documented while the experiments are carried out.

#### 3.5.2. Engineering of Additional Time Series Variables

Several quotient features, i.e., features that are constructed as the quotient of other features, are constructed manually and added to the data set. This is particularly useful because the machine learning algorithm used in this work, random forest, is known to show bad performance at modeling these types of features [38].

(1) $$\mathrm{QuotientPressureAxisX}1=\frac{\mathrm{PressureAxisX}1\mathrm{SideA}}{\mathrm{PressureAxisX}1\mathrm{SideB}}$$

(2) $$\mathrm{QuotientPressureAxisX}2=\frac{\mathrm{PressureAxisX}2\mathrm{SideA}}{\mathrm{PressureAxisX}2\mathrm{SideB}}$$

(3) $$\mathrm{QuotientPressureAxisX}3=\frac{\mathrm{PressureAxisX}3\mathrm{SideA}}{\mathrm{PressureAxisX}3\mathrm{SideB}}$$

(4) $$\mathrm{QuotientPressureAxisZ}1=\frac{\mathrm{PressureAxisZ}1\mathrm{SideA}}{\mathrm{PressureAxisZ}1\mathrm{SideB}}$$

#### 3.5.3. Feature Extraction Using the Methodology of the Randomized-Supervised Time Series Forest

Due to the multisensor approach and the various variables added by the machine’s PLC as well as the engineered time series variables, each forming process is represented by a multivariate time series. The direct use of such a data set for machine learning is not reasonable due to the high dimensionality (in the present application, there are over 30,000 dimensions when using each measurement point of each time series variable as a single dimension) and the strong correlations between dimensions [17]. Moreover, such a view of the data ignores its time series nature, i.e., the fact that the data points have a defined temporal sequence. To reduce this high number of dimensions and to capture the temporal characteristics of the data, a feature extraction algorithm based on the randomized-Supervised Time Series Forest (r-STSF) is implemented.

The r-STSF is a machine learning algorithm for time series data described in [8] as an advanced successor of the Time Series Forest [39] and the Supervised Time Series Forest [40]. In the area of time series classification, the r-STSF achieves the state-of-the-art performance of complex and computationally expensive algorithms such as HIVE-COTE (Hierarchical Vote Collective of Transformation-Based Ensembles, see [41]) while being several orders of magnitude faster [8]. A feature extraction algorithm, which generates a pool of interval features from a set of time series data, is a key aspect of r-STSF.

The features generated by this feature extraction algorithm are interval features, i.e., features which are calculated by applying an aggregation function, such as the mean or the standard deviation on a consecutive subset of the values of a time series. Interval features are robust to shifts of the time series on the time axis and are able to capture the temporal characteristics of the data. Also, they are quick to calculate and intuitively interpretable. Intervall features can be calculated on the raw time series data as it is, but just as well on other time series representations, i.e., transformed versions of the time series. The feature extraction algorithm of r-STSF uses four time series representations, the raw time series, the frequency domain representation (calculated with discrete Fourier transformation), a derivative representation (first order difference of the time series) and an autoregressive representation (autoregression coefficients).

Algorithmically, the feature extraction is based on a recursive search for suitable interval features on successively restricted intervals within the time axis of the data set. The time series of the training data set are first divided into a left subset and a right subset at a randomly chosen point. Both subsets are then recursively subdivided randomly again and again until no further subdivision is possible. In each iteration of this recursion, a selected aggregation function, such as the arithmetic mean or the standard deviation, is applied to both subsets created during the subdivision, and the resulting interval feature is rated. As an estimation of the usefulness of each given interval feature for the classification task, the Fisher score (see [42]) is used as the metric for this rating. Of two interval features created at one devision, only the feature with the better score is included in the feature pool, and the recursion is continued only for the interval whose feature has the better score. This procedure is repeated for the chosen sets of aggregation functions and time series representations.

Due to the promising quality of the features for the reasons mentioned above as well as the speed of computation, this method is used as a basis for the feature extraction algorithm implemented in the present work, hereafter called Supervised Time Series Feature Generation or STSFG. Speed is an important criterion especially because of the large number of time series variables. By implementing the feature extraction functionality autonomously (instead of using the complete r-STSF algorithm), the following advantages are achieved:

- A feature selection can be incorporated between the feature extraction and the training of models on the generated features (see Figure 14).
- The Extra Tree algorithm is an integral part of the r-STSF as defined by [8]. By detaching the feature extraction functionality from the rest of r-STSF, it becomes possible to train arbitrary other machine learning algorithms on the generated features.
- In addition to the choice of learning algorithm, the choice of libraries and implementations used for it is also open (Cabello et al. [8] use the Python library sklearn). In the present work, the machine learning platform H2O is used to train the models.

Furthermore, the implemented STSFG algorithm integrates modifications and extensions to handle multivariate time series and metric target variables. Multivariate time series are not considered in [39,40], or [8], although such extensions are held out in prospect for future work in the latter two cases. The algorithm implemented here handles multivariate time series by using an additional loop over all time series variables and accumulating all the resulting features in the feature pool.

Due to the fact that some of the target variables to be predicted in the given application are metrically scaled, there is a need to be able to perform feature extraction for such target variables as well. Since the Fisher score proposed in [8,40] is only defined for classifications, Kendall’s tau is used instead. As a rank correlation coefficient, Kendall’s Tau is nonparametric, robust to outliers [43], and can also detect nonlinear correlations as long as they are monotonic [35]. Compared to Spearman’s Rho, it is more robust and efficient [43].

The STSFG algorithm is implemented entirely in R. The pseudocode in Algorithms 1 and 2 describes the implementation, omitting certain implementation details for clarity, but without changing the logic of the algorithm.

**Figure 15:**
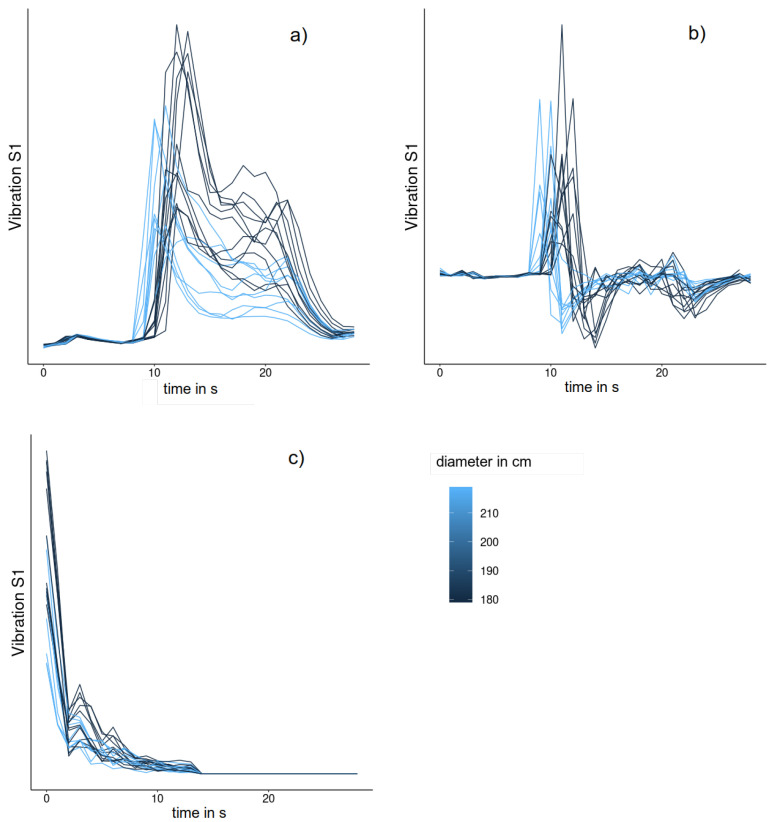
Vibrations of the spindle on three different time series representations. For a subset of the experiments, the figures show the strength of the vibration at the spindle (without unit) over time as raw time series data (**a**), in the time series representation of the derivatives (**b**) and at the frequency level (**c**). Each line represents a single experiment and the color of a time series depicts the diameter of the blank used. The sensor outputs values on a sensor-specific unitless scale, hence no labels are given on the y-axis.

In addition to the raw time series data, the derivative representation and the frequency domain representation are used. The autoregressive representation is not included yet but could be added without further modifications to the algorithm. Figure 15 illustrates different time series representations of the variable *VibrationS1*, which represents the strength of vibration at the spindle of the machine during the forming process. The aggregation functions used are the maximum and sum for binary features and the arithmetic mean, standard deviation, maximum, and root mean square (RMS) for metric features. The standard deviation is extended according to [39] so that it is also defined for intervals with only one element, in which case it is set to zero.

The STSFG algorithm has only one parameter, ${\mathrm{stsfg}}_{r}$, which is the number of repetitions performed. Cabello et al. [8] investigate this parameter’s influence in the r-STSF (called *d* there) and ultimately recommend setting it to 50 by default [8]. However, since in the STSFG the whole algorithm is additionally repeated for all time series variables, a parameter setting of ${\mathrm{stsfg}}_{r}=50$ leads to extremely long run times and feature sets that are no longer manageable due to their size. Therefore, the value ${\mathrm{stsfg}}_{r}=5$ is used here instead.
**Algorithm 1:** Supervised Time Series Feature Generation: Repeated iteration over all time series variables and time series representations.  **Input**: *X*: Set of |I| time series with |P| measuring points und |V| variables; *y*:     Vector of labels of the |I| time series; *A*: Set of aggregation functions; $f_{r}$:     Rating function for features; $n_{repeats}$: Number of repetitions (*d* in [8])
  
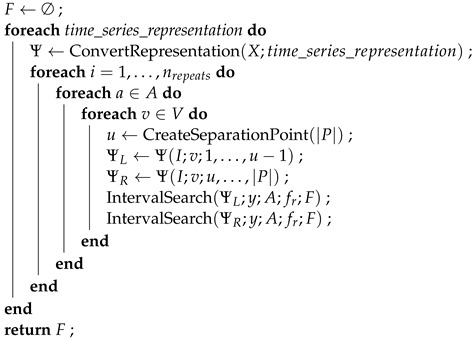


**Algorithm 2:** IntervalSearch: Generation of interval features on a time series variable in a time series representation.
  
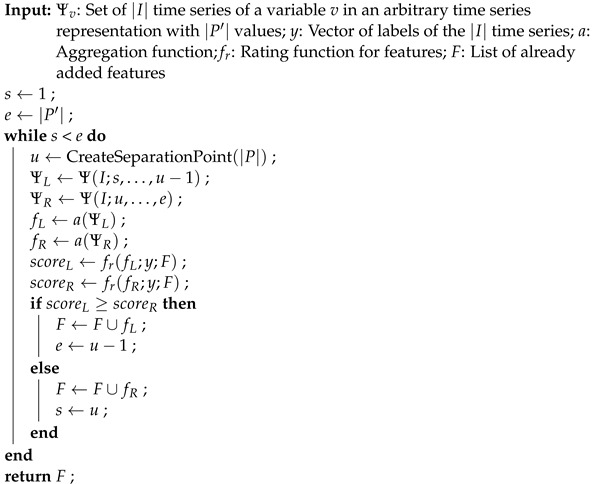



#### 3.5.4. Feature Selection Using Recursive Feature Elimination

The feature sets obtained from feature extraction are very large, usually several thousand features, and also suboptimal in terms of redundancy and relevance. This is primarily due to the fact that the time series variables on which the feature extraction is based already contain redundancy and many are only slightly relevant or not relevant at all for the individual target variables. Therefore, the next step in the preprocessing procedure is feature selection. In this step, the previously extracted set of features is reduced to a subset with as little redundancy and as high relevance as possible. This is intended to achieve:

- Improvement of the predictive ability of machine learning models trained on the resulting feature set. The experiments of [7] suggest that this is also possible for random forests, which are generally considered to be robust against irrelevant features.
- Minimization of the required sensors.
- Simplification of the models and thus improvement of their interpretability.
- Reduction in the time required to train the machine learning models.
- Reduction in the time required for the evaluation of new observations. This is particularly relevant for a possible live monitoring of the forming processes, where the time available for an evaluation of new data is limited.

For feature selection, a Recursive Feature Elimination (RFE) algorithm is implemented, which recursively trains random forest models and computes the permutation importance of the features to drop the least relevant ones. The comparison of the feature sets and the final selection of the best feature set is carried out using the out-of-bag errors of the random forests. The programming language used is R. For the model training and the calculation of permutation importances, functions of the software H2O are called.

RFE algorithms can be used effectively against both redundant and irrelevant features within a data set. Moreover, as, for example [44,45] show, they are also suitable for cases where the number of features is in the four-digit range or above and far exceeds the number of observations. However, the results of [45,46] also suggest that not too many features should be removed at once. In this way, the computational effort for the RFE increases, but this is less significant due to the rather small number of observations in the present use case—especially since the subsequent generation of the machine learning models (including the hyperparameter optimizations) is, in turn, considerably accelerated by the reduced number of features.

The parameter ${\mathrm{rfe}}_{r_{perc}}$ determines how many features are to be removed per iteration of the RFE. For this parameter, the settings used in the literature differ widely. While in [47], only a single feature is removed per iteration (the simultaneous removal of multiple features is, however, mentioned as a possible option, see [47]), in other cases, up to 50 % of features are removed from the data set per RFE iteration (see, e.g., [44,46]). To keep the aforementioned risks of simultaneously eliminating too many features, a rather low value of ${\mathrm{rfe}}_{r_{perc}}=5\%$ is used here.

The parameters of the random forests only have small influence on the RFE [44,46] and care should only be taken that ${\mathrm{rfe}}_{n_{\mathrm{tree}}}$ is large enough for the metrics considered to stabilize sufficiently [46]. Therefore, these parameters are assigned default values from the literature.

#### 3.5.5. Model Generation and Selection

In order to monitor the forming processes and the condition of the machine, machine learning models are trained that take all data gathered during a forming process as input to predict the influencing factors of this process. In principle, arbitrary machine learning models can be trained on the feature sets generated with STSFG and RFE. However, as, e.g., shown by the experiments in [47], the selection of the machine learning algorithm has far less impact on the predictive ability of the trained models than the quality of the features. Various works in the field of data-driven condition monitoring also suggest that the selection of the learning algorithm has a comparatively low relevance in the present use case (see Section 2.1). Therefore, only random forests are used here for the prediction of the influence variables. The main reasons for choosing the random forest algorithm are as follows:

- Random forests usually have a good predictive ability, which depends only little on the settings of the hyperparameters.
- Due to the small size of the training data set and the high number of dimensions, there is a high risk of generating overfitted models. Random forests are largely immune to this.
- The RFE algorithm used also employs random forests. Thus, the selected feature set is to some extent tuned for random forests.

According to the recommendation of [8], the trees of the random forests are trained with the Extra Tree algorithm. To optimize the parameter $m_{\mathrm{try}}$, a grid search is employed in each run of the training pipeline (following the recommendation of [7], five values between 2 and the number of features are tested at even intervals). The number of trees $n_{\mathrm{tree}}$ is set to 2000 for all of the random forests. To generate the random forest models, functions of H2O are called.

## 4. Results

### 4.1. Evaluation Method

The methodology presented on Figure 14 is evaluated for all six of the factors present in experimental design II. In order to obtain estimators for the respective generalization errors that are as unbiased as possible, repeated k-fold cross-validation is used as the validation method. *k* is set to k=10, following the recommendation of [48]. The number of repetitions is set to 5, which is comparatively small, to keep the total running time within acceptable limits. This is necessary because both feature extraction and feature selection are extremely computationally expensive and the entire procedure is to be evaluated for the six target variables. To avoid bias in the error estimates obtained with cross-validation, the whole process from initial preprocessing of time series variables to model generation and selection is repeated in its entirety for each partitioning within the cross-validation procedure. Without this, the supervised feature extraction, STSFG, as well as the feature selection with RFE would introduce a selection bias to the error estimation [46].

The parameters of RFE and STSFG are not optimized here, but are given default values (see Section 3.5.3 and Section 3.5.4). Table 1 summarizes the parameter settings used for the RFE, STSFG and the not optimized parameters of the random forests.

### 4.2. Evaluation of Regression Models

For the regression models, the metrics RMSE, MAE, and $q^{2}$ are given. According to [49], $q^{2}$ is calculated from the MSE of the prediction and “SS, the sum of squares of the differences between observed values […] and their mean […]” in the validation data with

(5) $$q^{2}=1-\frac{MSE}{SS}=1-\frac{\sum_{i=1}^{N}{\left({\hat{y}}_{i}-y_{i}\right)}^{2}}{\sum_{i=1}^{N}{\left(y_{i}-\bar{y}\right)}^{2}}$$

Here, *y* is the vector of target variable values from the validation data, $\bar{y}$ is their mean, and $\hat{y}$ is the vector of predictions for *y*. $q^{2}$, unlike RMSE and MAE, does not have a unit, which is why it is useful as a metric to compare the results for different target variables. According to Equation (5), $q^{2}=0$ corresponds to a model that always predicts the target variable’s mean from the validation data, and $q^{2}=1$ corresponds to a model that makes perfect predictions and therefore has an MSE of 0.

For each of the metric target variables, Table 2 contains the estimate of the generalization error determined by cross-validation. In addition to the metrics RMSE, MAE and $q^{2}$, the standard deviation of each metric is given as a comparison value.

The cross-validation RMSE is substantially below the respective standard deviation for all metric variables. Especially for factor 1 (diameter of the blank), a very low RMSE is achieved in relation to the standard deviation, which also is reflected in the high $q^{2}$-value of 0.96. The models for factors 2 (thickness of the blank) and 6 (displaced hole in the blank) have average $q^{2}$ values of 0.85 and 0.61, respectively.

### 4.3. Validation of Classification Models

For each of the binary target variables, Table 3 contains estimates of the generalization error obtained by cross-validation. In particular, the metrics MCC (Matthews Correlation Coefficient), F1, precision, recall and accuracy are shown in this table. Advantages of the MCC are that it provides reliable results even in the presence of unbalanced class distributions and that it is symmetric with respect to a swap of class definitions [50].

Among the classification models, a substantially higher accuracy is achieved for factors 7 (defined deformation) and 10 (damaged roller) than for factor 9 (flow rate of cooling liquid) in all metrics considered. The average MCC is 0.81 for factor 10, 0.76 for factor 7 and 0.45 for factor 9.

### 4.4. Dimensionality Reduction

Over 16,000 of features are generated by the STSFG per target variable. Using the RFE, these large feature sets are reduced to less than 40 features on average. Table 4 shows, for each of the target variables, the average number of features before and after the RFE, as well as the percentages by which the feature sets are reduced on average.

## 5. Discussion

The analysis of the state of research has shown that the data-driven monitoring of flow-forming processes has so far been a research gap. While similar manufacturing processes have been already well researched in this respect, it has not been clear whether such monitoring can be implemented for flow-forming processes, which are considered to be very complex. Therefore, one of the key findings of this work is that it is possible to detect unacceptable conditions in flow-forming processes using data-driven condition monitoring based on sensor data. Another finding is that the methodology used in the present work, including the data acquisition with DoE and the data preprocessing, is a promising way to approach such problems.

For companies using flow forming machines, this opens up the potential to optimise production through the implementation of automated process monitoring systems. By providing timely warnings, recommendations, or even halting the machine when necessary, these systems can significantly reduce the risk of financial losses associated with defective output and wasted material. Consequently, companies that manufacture these machines can gain a competitive advantage by equipping their machines with data-driven automatic monitoring capabilities, thereby providing their customers additional value.

The feature extraction method STSFG, which was derived from the r-STSF algorithm, has proved to be a promising means for generating features from multidimensional time series data in the present use case. Furthermore, it has been shown that a subsequent RFE can massively reduce the number of features needed for a prediction, which, among other things, reduces the training time and enables faster predictions. The latter is particularly relevant in the context of live monitoring of the forming processes. Also, the combination of STSFG and RFE may not only be useful for monitoring flow-forming processes. It stands to reason that this approach for generating and selecting features from time series data may also be an option to consider for monitoring other machines and processes.

Even apart from applications in the context of any kind of condition monitoring, to the author’s knowledge, no work has yet been carried out that uses the still-very-young r-STSF algorithm in any practical application. The present work contributes to filling this gap and demonstrates the potential of this algorithm. The adaptation to multivariate time series and regression problems also expands the possible application areas. The implementation as a stand-alone feature extraction simplifies the integration into existing structures.

Nonetheless, the research conducted in the present work has several limitations that warrant acknowledgment. Firstly, the methodology used is rather complex, which may hinder reproducibility. Simplifying the methods could enhance accessibility and facilitate broader application. Secondly, only one example flow-forming process was examined, potentially limiting the generalizability of the findings to other instances of this manufacturing technique. Additionally, the accuracy of the models may not be sufficient depending on the specific application, highlighting the need for further validation in different contexts. Furthermore, as only specific defects were studied, it remains unclear how other defects may influence model predictions or whether general fault detection is feasible. Moreover, no satisfactory results were obtained for the influencing factors that were dropped after the first series of experiments, and the results for influencing factor 9, the flow rate of the cooling liquid, were less conclusive and require additional confirmation.

## 6. Future Work

While the present work fills research gaps, new research needs arise at the same time. Flow-forming processes have opened up a new area of application for data-driven process and machine monitoring. This offers various opportunities for further research. These include, in particular, the investigation of other, possibly more complex, flow-forming processes in order to examine to what extent the knowledge gained from the example flow-forming process can be transferred to other processes. Furthermore, ways to improve the prediction accuracy should be explored, e.g., by using a larger training data set or by implementing and comparing it with other methodologies and algorithms. For example, Cabello et al. [8] have already announced their own extension of r-STSF for multivariate time series (see [8,40]), the accuracy and computational efficiency of which could be compared to the approach used in the present work. Another approach to improve the prediction accuracy could be the utilization of additional sensors. For example, in [4], an ultrasonic monitoring system was demonstrated to be able to "to record changes in the tool-workpiece contact area, detect internal fractures in the part, and measure the thickness of parts spun in free air", although it had some limitations that may limit its practical applicability.

In the present work, the parameters of the used feature extraction and feature selection like ${\mathrm{stsfg}}_{r}$ and ${\mathrm{rfe}}_{perc}$ were assigned default settings and not investigated in more detail. Optimization of these parameters as well as a sensitivity analysis may provide further insight into the influence of the parameters here and further improve the predictive ability of the models. A possible improvement of the prediction accuracy with the help of learning algorithms other than Extra Trees should also be investigated. The same applies to the use of the autoregressive time series representation and other aggregation functions not yet adopted here from the r-STSF.

Beyond the mere monitoring of the current state, research into the prediction of defects of the machine is also conceivable, e.g., to determine the time until a machine component fails (“Remaining Useful Life” [21]). Such systems, which belong to the research area “Predictive Maintenance”, require a more comprehensive data acquisition, since the algorithms must be enabled to detect the development of a defect before it manifests itself [51].

Lastly, further research is also needed with respect to the r-STSF and the STSFG method derived from it in the present work. Future work can test these algorithms in other application areas and further investigate their potential for different production processes and for time series classification in general.

## Figures and Tables

**Table 1:** Parameters of the STSFG, the RFE and the random forests.

Parameter	Description	Value
${\mathrm{stsfg}}_{r}$	Repetitions of the STSFG	5
${\mathrm{rfe}}_{perc}$	Proportion of features removed in each RFE iteration	10%
${\mathrm{rfe}}_{n_{\mathrm{tree}}}$	Number of trees of the random forests trained in the RFE	1000
${\mathrm{rfe}}_{m_{\mathrm{try}}}$	Number of randomly selected features at each node of the random forests built in the RFE	$\sqrt{m}$, $\frac{m}{3}$
$n_{\mathrm{tree}}$	Number of trees of the random forests	2000

**Table 2:** For each target variable, the average RMSE, MAE, and $q^{2}$ on the validation sets of the 10-fold cross-validation with 3 replicates are given. Also given are the standard deviations of the variables, which serve as reference values.

Target Variable	Ø RMSE	Ø MAE	Ø $q^{2}$	Standard Deviation
influencing factor 1 (diameter of the blank)	2.74 mm	1.57 mm	0.96	14.88 mm
influencing factor 2 (thickness of the blank)	0.21 mm	0.13 mm	0.85	0.66 mm
influencing factor 6 (displaced hole in the blank)	1.20 mm	0.86 mm	0.61	2.20 mm

**Table 3:** Evaluation of the models for the binary target variables. The average MCC, F1, precision, recall and accuracy values on the validation sets of the 10-fold cross-validation with 3 repetitions are given.

Target Variable	Ø MCC	Ø F1	Ø Precision	Ø Recall	Ø Accuracy
influencing factor 7 (defined deformation)	0.76	0.89	0.81	0.92	0.91
influencing factor 10 (damaged roller)	0.81	0.87	0.82	0.94	0.93
influencing factor 9 (flow rate of cooling liquid)	0.45	0.72	0.66	0.82	0.72

**Table 4:** Evaluation of the dimensionality reduction by the RFE.

Target Variable	Ø Feature Count after STSFG	Ø Feature Count after RFE	Reduction
Influencing factor 1 (diameter of the blank)	16,719.7	21.1	99.87%
Influencing factor 2 (thickness of the blank)	17,113.3	33.0	99.81%
Influencing factor 6 (displaced hole in the blank)	16,787.8	18.6	99.89%
Influencingfactor 7 (defined deformation)	16,541.4	37.2	99.78%
Influencing factor 9 (flow rate of cooling liquid)	9294.3	30.0	99.68%
Influencing factor 10 (damaged roller)	16,329.6	12.7	99.92%

## Data Availability

Data is contained within the article.

## Abbreviations

The following abbreviations are used in this manuscript:

CBM	Condition-based Maintenance
CM	Condition Monitoring
CNC	Computerized Numerical Control
DoE	Design of Experiments
FEA	Finite Element Analysis
HIVE-COTE	Hierarchical Vote Collective of Transformation-Based Ensembles
LASSO	Least Absolute Shrinkage and Selection Operator
MAE	Mean Absolute Error
MCC	Matthews Correlation Coefficient
PLC	Programmable Logic Controller
r-STSF	Randomized-Supervised Time Series Forest
RFE	Recursive Feature Elimination
RMS	Root Mean Square
RMSE	Root Mean Square Error
RUL	Remaining Useful Life
STSF	Supervised Time Series Forest
STSFG	Supervised Time Series Feature Generation
SVM	Support Vector Machine
TCM	Tool Condition Monitoring
TSF	Time Series Forest

## Appendix A

**Table A1:** List of relevant measured variables.

Component	Measured Variable
spindle	revolutions per minute
	active current
	active torque
	active motor temperature
	vibration
feed of spindle	position
	position error
	pressure side A
	pressure side B
feed of roller 1 position	position error
	pressure side A
	pressure side b
	force side A
	force side B
	bearing temperature
feed of roller 2	position
	position error
	pressure side A
	pressure side b
	force side A
	force side B
	bearing temperature
feed of roller 3	position
	position error
	pressure side A
	pressure side b
	force side A
	force side B
	bearing temperature
tailstock traverse	pressure
	temperature traverse bearing
	force
	vibration
ejector	pressure
	force
hydraulic power unit	fill level
	temperature
	pressure
bearing lubrication spindle	fill level
	temperature
bearing lubrication tailstock traverse	fill level
	temperature
